# Does the breast reconstruction method have an impact on time delay to adjuvant chemotherapy – A comparison between autologous and expander/implant breast reconstruction

**DOI:** 10.1016/j.jpra.2022.06.001

**Published:** 2022-06-23

**Authors:** Monika Lanthaler, Katharina Spechtler, Johanna Krapf, Daniel Egle, Michael Sieb, Christoph Tasch, Rossella Spinelli, Gerhard Pierer, Thomas Bauer

**Affiliations:** a Department of Plastic, Reconstructive and Aesthetic Surgery, Innsbruck Medical University Hospital, Anichstrasse 35, Innsbruck 6020, Austria; b Department of Gynecology and Obstetrics, Innsbruck Medical University Hospital, Austria; c Tyrolean Robot Laboratory, Innsbruck, Austria

**Keywords:** Breast reconstruction, Adjuvant chemotherapy, Delay

## Abstract

**Introduction:**

This study aims to analyze whether autologous breast reconstruction as compared to expander/implant reconstruction has a higher risk of postoperative wound healing problems (WHPs) and thus potentially delays chemotherapy start.

**Methods:**

Between January 2012 and December 2019, a total of 64 women with NSME/SSME and autologous (Group1, *n* = 33) or expander/implant reconstruction (Group2, *n* = 31) and adjuvant chemotherapy were enrolled in this study conducted at Innsbruck Medical University Hospital. Immediate postoperative WHPs in each group were compared, and the time from operation to initiation of chemotherapy was analyzed. If the start of chemotherapy was postponed for more than six weeks postoperatively due to WHP, it was defined as delayed. Statistical analysis was performed with SPSS and Fisher's exact test.

**Results:**

More postoperative WHP occurred in Group 1 than in Group 2 (51.6% vs. 9.7%, *p* < 0.001). Due to WHP, chemotherapy start was delayed for more than six weeks postoperatively in 30.3% of Group 1 patients and 3.2% of Group 2 patients. Only small differences in age (Group 1: 47±1 vs. Group 2: 46±2 years) and BMI (Group 1: 24.3 ± 0.6 vs. Group 2: 23.3 ± 0.7 kg/m^2^) were found.

**Conclusion:**

Our study shows a far smaller risk for postoperative WHP and delay of chemotherapy start in the expander/implant group in comparison with the autologous group. In some selected patients with high urgency for adjuvant chemotherapy, a bridging operation by means of expander reconstruction prior to chemotherapy could be an oncologically safe pathway.

## Introduction

It is essential that breast reconstruction be oncologically safe and not increase the risk of relapse or hinder adjuvant chemotherapy by causing complications.^1^ Implant-based breast reconstruction is a safe method with low morbidity, low complication rate, and short operative time. Flap reconstruction is a longer operation with possible donor site complications. However, flap reconstruction is known to have a lower complication rate during adjuvant radiotherapy and better long-term esthetic outcome.^2^, ^3^, ^4^

Initiation of adjuvant chemotherapy within six to 12 weeks postoperatively is recommended by guidelines.^5^ In the Netherlands, six weeks is the maximum time aimed between surgery and initiation of adjuvant chemotherapy, as recommended by the European Society for Medical Oncology (ESMO)^6^ and the Netherlands Society for Plastic Surgery.^7^ Several studies^8^^,^^9^ have reported that delayed initiation of adjuvant chemotherapy is associated with lower overall and recurrence-free survival.

This study aims to investigate whether there is a difference in time delay to adjuvant chemotherapy due to postoperative wound healing problems (WHPs) between autologous (DIEP: deep inferior epigastric perforator flap, PAP: profunda artery perforator flap) and expander/implant breast reconstruction. We assumed that the more complex autologous reconstruction, which may be associated with a higher risk for complications, may in some cases delay the initiation of chemotherapy. Our time period, within which chemotherapy should be started, was six weeks postoperatively.

## Patients and methods

Between January 2012 and December 2019, a total of 64 women underwent skin-sparing mastectomy (SSME)/nipple-sparing mastectomy (NSME) with immediate breast reconstruction and consecutive adjuvant chemotherapy at our institution. The study was conducted at Innsbruck Medical University Hospital, Department of Plastic Surgery and Department of Gynecology and Obstetrics.

Of these 64 patients, 33 underwent autologous reconstruction (AR, Group 1), (Group 1a: DIEP, *n* = 29 (87.9%); Group 1b: PAP, *n* = 4 (12.1%)). The other 31 patients (Group 2) had a one-stage direct-to-implant reconstruction (IR, Group 2a: *n* = 14, 45.2%) or a two-stage expander/implant reconstruction (ER, Group 2b: *n* = 17, 54.8%). In the expander/implant group, we inserted the implant in a submuscular layer and used the Tiloop®Bra (pmf medical), which was sutured inferiorly to the pectoralis major muscle.

The mean age in the autologous group was 47 (± 1) years (DIEP: 47±2 years, PAP: 44±6 years). The expander/implant group had a mean age of 46±2 years (expander: 47±2 years, implant: 46±3 years). The mean BMI in the flap group was 24.3 ± 0.6 kg/m^2^ (DIEP 24.7 ± 0.7 kg/m^2^, PAP 21.4 ± 0.5 kg/m^2^), whereas the BMI in the expander/implant group was slightly lower with a mean BMI of 23.3 ± 0.7 kg/m^2^ (expander: 22.8 ± 0.9 kg/m^2^, implant: 24±1.1 kg/m^2^). All patients included in this study needed postoperative adjuvant chemotherapy due to tumor pathology.

### Statistical analysis

All data were retrospectively collected exclusively from our hospital's internal patient data system. Statistical analysis was performed with IBM SPSS version 27 and Fisher's exact test.

## Results

### Autologous breast reconstruction

Of the 33 patients with autologous breast reconstruction, **17 patients (51.6%)** suffered documented **WHPs** *(*Tables 1 and 2*)*, whereas 16 (48.4%) had no problems.

**Table 1:** Autologous and expander/implant breast reconstruction and postoperative complications.

Autologous breast reconstruction (Group 1)			*n* = 33	100%
Wound healing problems (WHP)			***n*** **=** **17**	**51.6%**
Conservatively treated WHP without chemotherapy delay			*n* = 7	21.2%
	*Flap*	*Start of chemotherapy (weeks)*	*Localization of WHP*	*n*
	DIEP	< 6	Breast	4
	DIEP	< 6	Abdomen	1
	DIEP	< 6	Breast and abdomen	1
	PAP	< 6	Thigh	1
Relevant WHP with chemotherapy delay			n = 6	18.2%
	DIEP	16	Breast and abdomen	1
	DIEP	12	Breast	1
	DIEP	8	Breast	3
	PAP	8	Thigh	1
Start of chemotherapy despite WHP			n = 4	12.1%
	DIEP			3
		8 , >8	Breast and abdomen	2
		7	Abdomen	1
	PAP	16	Thigh	1
**Expander/implant reconstruction (Group 2)**			***n*** **=** **31**	**100%**
**Wound healing problems**			***n*** **=** **3**	**9.7%**
WHP with chemotherapy delay			*n* = 1	3.2%

**Table 2:** Groups 1 and 2 – TTC.

		n	Ø time to chemotherapy TTC (weeks)	Ø BMI (kg/m^2^)
**Group 1**		33	6	24.3 ± 0.6
	Conservatively treated WHP	7	5	22.1
	Relevant WHP	6	11	25.6
	Chemotherapy despite WHP	4	7	27.3
**Group 2**		31	4	23.3 ± 0.7
	Conservatively treated WHP	2	5	22.2
	Relevant WHP	1	9	25.7

Of these 17 patients, **seven** (21.2% of the 33 flap patients) with a mean age of 49±2 years had **minor WHP that were resolved within six weeks postoperatively; thus, they commenced chemotherapy without any delay.** Initiation of chemotherapy in this group of patients started after a mean of 5 ± 0 weeks. Of these seven patients, four DIEP patients had WHP of the breast, one DIEP patient of the abdomen, and one further DIEP patient of the abdomen and breast. The PAP patient suffered thigh donor site WHP.

In **six patients** (18.2% of the 33 flap patients), the **initiation of chemotherapy was delayed due to WHP** with a mean time to chemotherapy of 11±1 weeks from the time of operation (two patients ≥12 weeks). The mean age was 51±4 years. Five patients had WHPs in the breast and one patient in the thigh following PAP.

In the other **four** (12.1% of the 33 flap patients) of the 17 patients with WHPs, **chemotherapy had to be started despite WHP** due to aggressive tumor biology and therefore the urgent need to start. The mean time for chemotherapy in this group was 7 ± 1 weeks. The mean patient age was 46±3. Two patients had WHP in the abdomen and breast, one in the abdomen, and one in the thigh region. Despite WHP, initiation of chemotherapy resulted in prolonged wound healing during chemotherapy, although in only two patients for more than 12 weeks.

### BMI and complications

**The seven patients** with WHP without any delay in commencing chemotherapy had a mean BMI of 21.5 ± 0.7 kg/m^2^.

**The six patients (DIEP: 5P, PAP: 1P**) with WHP and a mean chemotherapy delay of 11±1 weeks had a BMI of 25.6 ± 1.5 kg/m^2^.

The two DIEP patients with a BMI of 22.4 kg/m^2^ and 24.4 kg/m^2^ with a chemotherapy delay of about two months suffered WHPs in the breast. The PAP flap patient with also two months delay had WHPs in the thigh and a BMI of 21.9 kg/m^2^. The two further DIEP patients with a delay of about three months also suffered breast WHPs and had a BMI of 27.9 kg/m^2^ and a BMI of 25.5 kg/m^2^. One DIEP flap with a delay of about four months had a BMI of 31.4 kg/m^2^ and WHPs in the breast.

**The other four patients (3 DIEP, 1 PAP), who underwent chemotherapy despite existing WHP** because of the urgent need to start, had a mean BMI of 27.3 ± 2.8 kg/m^2^. The three DIEP flap patients had a BMI of 34 kg/m^2^ (WHP breast and abdomen), 29.6 kg/m^2^ (WHP abdomen), and 23 kg/m^2^ (WHP breast and abdomen). The PAP flap with WHP in the thigh had a BMI of 22.5 kg/m^2^.

The two PAP patients with WHP with a BMI of 21.9 kg/m^2^ and 22.5 kg/m^2^ had a relatively low BMI in comparison with the DIEP flap group.

The mean BMI of all DIEP patients with WHP was 25.2 kg/m^2^, whereas, in the PAP group, BMI was 21.5 kg/m^2^. The mean BMI in Group 1a without WHP was 24.3 kg/m^2^ in comparison with 21.2 kg/m^2^ in Group 1b.

### Expander/implant breast reconstruction (ER/DIR)

Of the 31 patients with expander/implant reconstruction (Group 1a, direct to implant: 14 P, 45.2%; Group 2b, expander: 17 P, 54.8%), **three patients (9.7%)** had postoperative **WHPs** (Table 2).

Of these three patients, **one (3.2%) patient** had a **chemotherapy delay of nine weeks** with a direct-to-implant reconstruction caused by a reductive incision NSME at the age of 45, and **two patients** at the age of 51±1 had **minor WHPs** in the breast with no delay in chemotherapy and chemotherapy initiation after a mean of 5 ± 1 weeks. No patient had to start chemotherapy despite WHP.

### BMI and complications

Mean BMI in Group 2a was 24 kg/m^2^, and in Group 2b was 22.8 kg/m^2^.

The direct-to-implant patient (3.2%) with the chemotherapy delay of nine weeks had a BMI of 25.7 kg/m^2^. The two patients with WHP and no delay in chemotherapy, which was initiated after a mean of 5 ± 1 weeks, had a mean BMI of 22.2 ± 2 kg/m^2^.

At 23.3 ± 0.7 kg/m^2^, the mean BMI in the expander/implant group was slightly lower than in the autologous group with a mean BMI of 24.3 ± 0.7 kg/m^2^.

## WHP autologous vs. expander/implant reconstruction

More postoperative complications occurred in Group 1 than in Group 2 (51.6% vs. 9.7%, *p* < 0.001). Due to WHP, chemotherapy was delayed for more than six weeks postoperatively in 30.3% of Group 1 patients and 3.2% of Group 2 patients. Only small differences in age (Group 1: 47±1 vs. Group 2: 46±2years) and BMI (Group 1: 24.3 ± 0.6 vs, Group 2: 23.3 ± 0.7 kg/m2) were found.

## Complications with necessary operative revision

Of the six patients with **flap reconstruction** and a delay in commencement of chemotherapy, five patients underwent operative revision. Despite WHPs, of the four patients who started chemotherapy, three patients had an operative revision. Of the seven patients who started chemotherapy within six weeks with WHPs beforehand, five patients had an operative revision. Thus, of the 17 patients with WHPs, **13** patients (76.5%) had to undergo operative revision. These make up **39.4% of the 33 flap patients**.

In the **expander/implant group, two patients (6.5%)** had an **operative revision,** both of whom underwent reductive incision site NSME/SSME with poor blood perfusion of the skin flap margins. One of these patients had a breast reduction ten years prior to reconstruction, and the other one had NSME in a reductional pattern.

## Operative corrections in the long term

In the long term, six (18.2%) of the 33 flap patients and six (19.4%) of the 31 expander/implant patients required operative correction.

In the **flap group,** 27 of the **33 patients did not need further correction** in the long term. The remaining **six patients** underwent **small contouring corrections** mainly by means of lipofilling and liposuction.

According to our electronic patient data system, **six of the 31 expander/implant patients** underwent **operative revision in the long term**. After radiotherapy, **two patients** presented late secondary WHPs with exposed implant. One of these patients underwent revision with the placement of a new expander and after six months re-implant reconstruction, whereas the other one switched to DIEP reconstruction. Both patients had further contouring operations with lipofilling. Of **two further patients** who underwent reconstruction by means of reductive incision, one switched to PAP reconstruction due to problems with the implant and one finally wanted a larger implant and was given a new expander. The **fifth patient** had WHP in the long term, which was resolved in a second successful expander/implant reconstruction. The **sixth patient** had lipofilling following direct-to-implant reconstruction.

## Discussion

**As described in the literature,** chemotherapy should be started within six to 12 weeks postoperatively.

In their retrospective study of 2.594 patients, Lohrisch et al.^10^ reported a decrease in disease-free and overall survival (OS) with a delay of adjuvant chemotherapy beyond 12 weeks post-surgery. Their results highlight the importance of timely administration of chemotherapy within three months of surgery whenever possible.

The data published by Colleoni et al.^11^ showed improved outcome in postmenopausal patients with ER-absent tumors with early initiation of systemic chemotherapy. Ten-year disease-free survival was 60% for the early initiation group (20 days, 599 patients) compared with 34% for the conventional initiation group (21–80 days, 1189 patients).

In their study of 6827 patients, Gagliato et al.^9^ reported poorer OS for stage III breast cancer patients when the initiation of chemotherapy started >60 days after surgery. Patients with triple-negative tumors and HER2-positive tumors treated >61 days after surgery had poorer survival than did those who initiated treatment in the first 30 days after surgery. According to them, especially in high-risk groups like patients with stage II and III tumors and HER2-positive tumors, every effort should be made to avoid postponing the initiation of adjuvant chemotherapy.

The meta-analysis by Biagi et al.^12^ that included data on 15.327 patients reported that each four-week delay in the initiation of adjuvant chemotherapy resulted in a 6% increase in the risk for death and an 8% increase in the risk for relapse.

In their study of 24.843 patients, Chavez-MacGregor et al.^8^ report poorer OS for patients undergoing chemotherapy >91 days after surgery with a 34% increase in the risk for breast cancer death. In particular, patients with triple-negative breast cancer had poorer OS.

Yu et al.^13^ identified 34.097 patients from seven different studies and observed that OS decreased by 15% for every additional 4-week delay in initiation of adjuvant chemotherapy. Thus, a 12-week delay would be associated with an approximately 30% increase in the risk for death.

In their study of 172.043 patients, Kupstas et al.^14^ report that chemotherapy commencement beyond 120 days after diagnosis resulted in poorer OS for all biological subtypes. In this group, the interval from diagnosis to surgery was delayed for reconstructive cases, but not the interval from surgery to chemotherapy. In this cohort, patients with breast-conserving surgery had a significantly shorter time from diagnosis to surgery (median 25 days) than did those who underwent tissue-based or implant-based reconstruction (median 35 days). The median time from surgery to chemotherapy was 43 days for all patients with a slightly longer interval of median 45 days for tissue-based and median 44 days for implant-based reconstruction. In this group, tissue-based reconstruction patients were slightly more likely to experience a delay in chemotherapy (16% versus 13% with >120 days from diagnosis to chemotherapy start).

As mentioned above, in the Netherlands, six weeks is the maximum time period aimed for between surgery and initiation of adjuvant chemotherapy, as recommended by the European Society for Medical Oncology^6^ (*EMSO*) and *the Netherlands Society for Plastic Surgery*.^7^^,^^8^ Several studies^9^^,^^10^ have reported that delayed initiation of adjuvant chemotherapy is associated with lower overall and recurrence-free survival. The recommended maximum delay varies from seven to 12 weeks.^5^

**Our study** shows more postoperative complications in Group 1 than in Group 2 (51.5% vs. 9.7%, *p* < 0.001). Due to WHP, chemotherapy start was delayed for more than six weeks postoperatively in 30.3% of Group 1 patients and 3.2% of Group 2 patients. Thus, our study shows a 10-fold higher risk for chemotherapy delay in autologous reconstructed patients. Nevertheless, we have to consider that of the six patients with chemotherapy delay and initiation of chemotherapy after a mean of 11±1 weeks from the time of operation, only two patients had a delay of ≥12 weeks. The four patients who started chemotherapy after a mean of 7 ± 1 weeks, despite WHP, of course, had prolonged WHP during chemotherapy, but these problems lasted more than 12 weeks in only two patients. Thus, it may also be possible to start chemotherapy, despite WHP, when it is urgently necessary.

As seen in our study and as also reported in the literature,^15^, ^16^, ^17^ autologous reconstruction, especially with a higher BMI, entails a higher risk for WHP due to large donor site incisions.

In our study, only a small difference in age (Group 1: 47±1 vs. Group 2: 46±2 years) and BMI (Group 1: 24.3 ± 0.6 kg/m^2^ vs, Group 2: 23.3 ± 0.7 kg/m^2^) was seen between our two groups. As seen from our data, patients with autologous reconstruction are slightly older, and especially the DIEP group had a slightly higher BMI.

We know from our clinical experience that younger, especially thin patients, more often opt for expander/implant reconstruction as they do not want additional scars.

Patients with a higher BMI often opt for the benefit of an additional abdominoplasty with the DIEP flap as reflected in the slightly higher BMI in this group. However, we also often offer DIEP flap reconstruction to relatively thin patients and they sometimes have WHPs in the abdomen due to wound closure tension, as we nevertheless try to place the scar low so that it is covered by underwear. Tension in these patients can sometimes be avoided only by placing the scar higher, but with the disadvantages of visibility and lesser flap volume. Contrary to this problem in thin patients, in adipose patients, WHPs often occur due to less blood supply to the fat tissue in the abdomen and breast. As adipose patients often opt for a DIEP reconstruction, this on the one hand raises the question of whether obese patients who want a DIEP reconstruction with an aggressive tumor biology that requires urgent chemotherapy should primarily in a first step be reconstructed with an expander and after completion of adjuvant chemotherapy undergo DIEP reconstruction. Another possible means of reducing WHP in obese patients may be to locate the abdominal scar higher if the patient shows an aggressive tumor as mentioned above. Nevertheless, obese patients also often tend to have increased WHPs in the breast due to reduced blood flow in the thick fat tissue.

Another possible explanation for the relatively high complication rate in our autologous breast reconstruction group may be the learning curve of different sometimes young oncologic surgeons operating at a university hospital such as ours. Reduced blood flow in the mastectomy flap could in some cases be a result of this learning curve with subsequent complications.

Furthermore, we should not forget the adverse risk factor of smoking, especially in these patients. We try to avoid flap reconstruction in heavy smokers and strongly recommend that such patients reduce tobacco use, although the urgency of tumor resection precludes any efforts to check on tobacco use. Also, in these patients, a possible reduction in WHP may be seen, when a primary expander reconstruction is performed, and after three months, if the patient has stopped smoking, autologous tissue reconstruction is offered.

As known from the literature, autologous breast reconstruction patients more often suffer from postoperative WHPs as a result of larger incision sites and the more complex operation. The implant/expander group presents with fewer complications immediately after surgery, whereas, in the long term, this group often needs more operative revisions. As seen from our study, the autologous group mostly needs only small contouring operations like lipofilling in the long term, whereas the implant group often needs major revisions. It is of great importance to clearly explain the advantages and disadvantages of each procedure to the patient preoperatively and to make the right decision together with the patient.

However, if postoperative chemotherapy is urgently indicated due to tumor pathology and patients are at a high risk for WHP, such as those with a high BMI, thorough interdisciplinary cooperation is necessary to avoid delaying chemotherapy. Perhaps in some cases with aggressive tumor pathology, primary expander reconstruction with secondary autologous reconstruction following chemotherapy can be a worthwhile oncologically safe pathway if no postoperative radiotherapy is planned. If radiotherapy is necessary, we feel the switch from expander to autologous reconstruction should be performed after chemotherapy and before radiation, if oncologically justifiable.

## Conclusion

In summary, our data show that the expander/implant group had fewer immediate postoperative WHP and thus less risk for a delay in chemotherapy start than did the autologous group, although only two patients had a delay of ≥12 weeks. Contrary to this, in the long term in the implant group, especially radiated patients undergo more revisions than autologous reconstructed patients, who mainly require only contouring corrections.

It is important to decide interdisciplinarily for each patient, especially for those with existing additional risk factors, which approach is the best choice with the best outcome, namely particularly in patients with highly aggressive tumors where the risk for WHP should be minimized. To avoid postponing chemotherapy, in selected cases, a bridging operation with primary expander reconstruction, following autologous reconstruction after chemotherapy, could be a possible oncologically safe treatment pathway.

*Addendum*

The study was conducted simultaneously as a Master's Thesis (Diplomarbeit) by a medical student (K. Spechtler) listed as coauthor. The thesis was supervised by the first author M. Lanthaler.

## Statement of ethics

This study protocol was reviewed and approved by the Ethics Committee of the Medical University of Innsbruck. (Votum: EK Nr: 1484/2020).

## Funding sources

Open access agreement Innsbruck Medical University. There is no other funding of any research relevant to this study.

## Declaration of Competing Interest

The authors have no conflicts of interest to declare.
